# Draft Genome Sequence of Methylovulum psychrotolerans Sph1^T^, an Obligate Methanotroph from Low-Temperature Environments

**DOI:** 10.1128/genomeA.01488-17

**Published:** 2018-03-15

**Authors:** Igor Y. Oshkin, Kirill K. Miroshnikov, Svetlana E. Belova, Aleksei A. Korzhenkov, Stepan V. Toshchakov, Svetlana N. Dedysh

**Affiliations:** a Winogradsky Institute of Microbiology, Research Center of Biotechnology RAS, Moscow, Russia; b Immanuel Kant Baltic Federal University, Kaliningrad, Russia

## Abstract

Methylovulum psychrotolerans Sph1^T^ is an aerobic, obligate methanotroph, which was isolated from cold methane seeps in West Siberia. This bacterium possesses only a particulate methane monooxygenase and is widely distributed in low-temperature environments. Strain Sph1^T^ has the genomic potential for biosynthesis of hopanoids required for the maintenance of intracytoplasmic membranes.

## GENOME ANNOUNCEMENT

Methylovulum psychrotolerans Sph1^T^ is a neutrophilic aerobic methanotroph of the class Gammaproteobacteria, family Methylococcaceae. It was isolated from a cold methane seep in the Irtysh basin, West Siberia (1). This methanotroph grows well at low temperatures and is commonly detected in various low-temperature environments, such as arctic lakes, glaciers, and northern peatlands (2–4). Cells of M. psychrotolerans Sph1^T^ are Gram-negative, nonmotile, encapsulated, large cocci that multiply by binary fission and utilize methane and methanol.

For the genome sequencing of strain Sph1^T^, a combination of fragment and mate-paired library approaches was used. Both libraries were sequenced with the Illumina MiSeq platform using 2 × 250-bp paired-end sequencing reagents. Mate-paired reads were treated with the NextClip tool (5), resulting in 1,082,354 read pairs with a mean insert size of 3,183 bp. Paired-end reads were filtered and trimmed by quality with CLC Genomics Workbench (Qiagen, Germany) using recommended parameters, resulting in 431,801 read pairs. *De novo* assembly was performed with the SPAdes version 3.11.0 assembler (6). The total length of the final assembly was 5,189,806 bp; it consisted of 97 genomic scaffolds with an *N*_50_ value of 334,445 bp. Final genome coverage was 40× for the mate-paired library and 23× for the paired-end library. The estimated size of the M. psychrotolerans Sph1^T^ genome is 5.2 Mb (coverage, 63×), with an average G+C content of 50.8%. In total, 4,577 predicted protein-coding genes were identified.

The average nucleotide identity between the genomes of M. psychrotolerans Sph1^T^ and another described member of this genus, M. miyakonense HT12^T^ (7), is only 79%, which confirms their classification as two different species. The genome of M. psychrotolerans Sph1^T^ contains a single *pmoCAB* operon for particulate methane monooxygenase (MMO). The presence of the homologous *pxmABC* operon, which is characteristic of many gammaproteobacterial methanotrophs (7, 8), was not detected. In contrast to M. miyakonense HT12^T^, an operon encoding soluble MMO (*mmoXYBZDC*) is lacking in M. psychrotolerans Sph1^T^. The ability to grow on methanol is explained by the presence of the gene operons encoding MxaFI- and XoxF-methanol dehydrogenases (9). Genes involved in tetrahydromethanopterin (H_4_MTP), tetrahydrofolate (H_4_-folate)-linked C1 transfer, and formate oxidation were identified. A complete set of genes encoding formaldehyde assimilation in the ribulose monophosphate pathway was present, while the serine cycle was incomplete.

The genome includes all genes required for glycogen biosynthesis (*glgA*, *glgB*, and *glgC*) (10) and nitrogen metabolism, including genes for nitrate/nitrite reduction, ammonium and urea uptake and assimilation, as well as key genes for nitrogen fixation.

Strain Sph1^T^ has the genomic potential for the biosynthesis of hopanoids, which is required for the maintenance of intracytoplasmic membranes (11). A complete set of genes for the nonmevalonate pathway and the *ispA* gene encoding farnesyl diphosphate synthase were identified. The genes responsible for squalene synthesis (12), two copies of the *shc* gene encoding the enzyme responsible for the cyclization of squalene into diploptene (13), as well as the *hpnH* and *hpnG* genes involved in the conversion of diploptene to bacteriohopanetetrol (14) were also present.

### Accession number(s).

The M. psychrotolerans Sph1^T^ genome sequence was deposited in GenBank under the accession no. PGFZ00000000.
